# Health-seeking behaviour during times of illness among urban poor women: a cross-sectional study

**DOI:** 10.1186/s12905-024-03178-w

**Published:** 2024-06-07

**Authors:** Khadijahtul Qubra Amizah Hamzah, Nor Afiah Mohd Zulkefli, Norliza Ahmad

**Affiliations:** Department of Community Health, Faculty of Medicine and Health Sciences, Universiti Putra Malaysia, Serdang, Selangor 43400 Malaysia

**Keywords:** Health-seeking behaviour, Urban poor, Women

## Abstract

**Background:**

Urban poor women face dual challenges regarding gender inequalities and urban poverty, which make them more likely to have health problems and affect their health-seeking behaviour. This study aimed to determine the prevalence of health-seeking behaviour during times of illness and predictors of sought care among urban poor women in Kuala Lumpur, Malaysia.

**Methods:**

This cross-sectional study was performed among 340 randomly selected women residents from April to May 2023. Data was collected using a validated and reliable self-administered questionnaire and analysed using SPSS version 28.0 software. The dependent variable in this study was health-seeking behaviour during times of illness, while the independent variables were sociodemographic characteristics, socioeconomic characteristics, medical conditions, women’s autonomy in decision-making, social support, perceived stigma, and attitude towards health. Multiple logistic regression was used to identify the predictors of sought care during times of illness.

**Results:**

Study response rate was 100%, where 72.4% sought care during times of illness. Being non-Malay (AOR = 4.33, 95% CI: 1.847, 10.161), having healthcare coverage (AOR = 2.60, 95% CI: 1.466, 4.612), rating their health as good (AOR = 1.87, 95% CI: 1.119, 3.118), and having pre-existing chronic diseases (AOR = 1.92, 95% CI: 1.130, 3.271) were identified as predictors of sought care during times of illness.

**Conclusion:**

The present study showed that health-seeking behaviour during times of illness among the participants was appropriate. Health promotion and education, with a focus on educating and raising awareness about the importance of seeking timely healthcare, are crucial to improving health-seeking behaviour among urban poor women. Collaboration with relevant stakeholders is needed to develop comprehensive strategies to improve access to healthcare facilities for these women.

## Background

Health and well-being are the basic needs of a human being. The United Nations, through its third Sustainable Development Goal (SDG), targets that everyone has access to the full range of high-quality healthcare services they require, when and where they need them, and without financial hardship [1]. This target, known as universal health coverage (UHC), encompasses all aspects of healthcare services, from health promotion to prevention, treatment, rehabilitation, and palliative care. The UHC initiative could be significantly impacted by the health-seeking behaviour (HSB) of the population [2]. HSB is “any action or inaction taken by individuals who perceive themselves to have a health problem or to be ill to find an appropriate remedy” [3]. HSB, or illness behaviour or sick-term behaviour, is part of the broader term of health behaviour [4]. Inappropriate HSB can impact population health outcomes [5], increase the community’s transmission risk, increase the disease burden and cause premature death [6]. By comprehending the patterns of HSB, public health practitioners and policymakers can improve the healthcare system and health promotion strategies [7].

Even though past studies showed that women seek health more than men [8, 9], women in developing countries use proper healthcare less than men and prefer traditional healing methods and self-medication [10]. These are considered inappropriate HSB [11]. According to Harrison and colleagues [12], the gender socialisation process, which is influenced by socio-cultural factors, has an impact on health-related notions and habits, such as decisions about when and where to seek care. Not only does it impact how symptoms or distress are expressed and treated, but it also often leads to other societal barriers that hinder women from obtaining the medical care they need. Women who lack knowledge about health issues downplay their illnesses or rely on older relatives or men to look after them. This becomes more evident in nations where structural barriers prevent women from seeking medical attention for their illnesses.

Women in urban areas have more opportunities and job options than women in rural areas. However, they live in an unfavourable environment because of gender inequality, high living costs, and vulnerability to violence, making them more likely to have health problems [13, 14] particularly those living in poverty [15]. Urban poverty is complex and multifaceted, extending beyond a lack of income or resources. The urban poor face issues such as inadequate housing, limited access to basic services, low income levels, and health burdens [15]. The urban poor often lack representation and voice in policy-making processes, contributing to their marginalisation [16]. The multiple pressures faced by urban poor women increase their health risks. However, women, especially impoverished women, have limited access to formal healthcare services for their health conditions, and sometimes the costs can be prohibitively high [14, 17]. Monitoring gender inequality is essential to identify and track disadvantaged populations to provide decision-makers with an evidence base to formulate more equity-oriented policies, programs and practices towards the progressive realisation of UHC.

The prevalence of HSB varies from illness to illness. HSB among women in Malaysia showed an increasing trend from 36.4% in 2011 to 60.1% in 2019 [18]. However, HSB in Malaysia is still low compared to the goal set under the United Nations UHC strategies, which targets access to quality essential healthcare services for everyone [1]. In the nationwide survey, HSB in Kuala Lumpur is among the lowest since 2011 (36.4%), 39.0% in 2015, and 49.6% in 2019 [18]. Kuala Lumpur is the federal city of Malaysia, with a wide distribution of public and private healthcare facilities compared to other states. In Malaysia, the prevalence of diseases among women showed an increasing trend for example diabetes increased from 9.1% (2015) to 9.8% (2019), hypertension from 14.0% (2015) to 17.8% (2019), and hypercholesterolemia from 9.8% (2015) to 15.1% (2019) [19].

A review of previous studies suggested that HSB among women could be associated with disease patterns, psychosocial factors, women’s autonomy, self-rated health, cultural beliefs, health literacy, and issues related to healthcare services. Urban poor women often seek healthcare services due to chronic diseases, mental health issues, and reproductive care needs [20, 21]. Psychosocial factors encompass psychological and social factors also affecting how women seek healthcare [5]. Psychological barriers such as stereotypes and stigma about certain health conditions or seeking healthcare can lead to negative emotions such as shame, fear, and embarrassment, preventing them from getting necessary healthcare on time [22]. Urban poor women challenging circumstances and limited resources make social support crucial for their HSB. Managing diseases successfully requires cooperation between patients, families, and healthcare professionals. For this reason, social support alleviates anxiety, reduces stigma, and provides reassurance, resulting in a more positive healthcare experience [23].

Moreover, women’s autonomy in healthcare decisions significantly impacts maternal and child health outcomes and reflects women’s empowerment. It also facilitates access to material and social resources such as food, income, knowledge, healthcare, and power within the family and community [24]. Self-rated health is a person’s subjective evaluation of their state of health. Particularly, people who rate their health as poor are more likely to seek medical attention when they experience illness-related symptoms [20, 25]. Additionally, cultural beliefs may influence perceptions of illness, treatment preferences, and attitudes towards healthcare providers, which demonstrates the strong influence of sociocultural practices and beliefs on one’s HSB [26, 27].

Furthermore, health literacy is a critical determinant of health-seeking behaviour, as those with better health literacy are more likely to participate in preventive health behaviours and effectively use health services [28]. Low health literacy has been linked to poor utilization of healthcare resources and poor health outcomes, especially among vulnerable populations [28, 29]. Above all, the healthcare system can also influence a woman’s HSB. When health services are far away, the likelihood of seeking care decreases, and vice versa [30]. This means that people who live farther away from healthcare services may need help accessing the care they need, as indirect costs incurred during travel may make services less affordable. Additionally, the availability and accessibility of healthcare services, including the operating hours of public health facilities, are crucial factors limiting women’s ability to obtain the care they need [31].

Available research in this field focused mainly on pregnancy and maternal health [21], mental health [22, 32] and other disease-related [33, 34]. Limited studies suggest that HSB during times of illness may be influenced by age, household income level, education level, and self-rated health [7, 25]. However, these studies were conducted in Ghana and Hong Kong and not among the urban poor women population. The findings have their limitations as there are also healthcare system and cultural differences between these countries and Malaysia.

Little is known about the behaviour of seeking healthcare among urban poor women during times of illness in Malaysia although there have been some studies conducted addressing this issue in other ethnicities such as in African women [25, 34]. Considering the lack of study on HSB during times of illness, therefore, the present study aimed to investigate the predictors of HSB during times of illness among urban poor women in Kuala Lumpur.

## Methods

### Study design and area

A population-based cross-sectional study was conducted using the STROBE guidelines from April to May 2023 in a people’s housing project in Kuala Lumpur, the federal city of Malaysia [35]. The city’s population density was estimated to be 8,157 people per square kilometre in 2020, making Kuala Lumpur the most populous city in Malaysia [36]. Kuala Lumpur recorded 193.7 thousand households with an income of RM9,149 or less, which is categorised as the low-income group [37]. The people’s housing project was an initiative by the Malaysian government during the 1997 Asian economic crisis to improve the national economy by providing affordable housing for low-income people [38]. There are about 30 people’s housing projects in Kuala Lumpur and The People’s Housing Project Seri Semarak was chosen due to its location in the city centre with an estimated 2000 women residents.

### Study population and sample size determination

All adult women residents aged between 18 and 59 years old with household incomes of RM9,149 and lesser, understood either Malay or English language with self-reported illness for the past three months before data collection were eligible to participate in the study. Women residents who were non-Malaysians, with special needs (mental, visual or hearing disabilities) or pregnant during data collection were excluded from the study. The sample size of this study was determined by using the two population proportions by Lwanga and Lameshow (1991) on the self-rated health variable from a previous study, considering P_1_ = 0.56, the proportion of HSB among good self-rated health, and P_2_ = 0.40, the proportion of HSB among fair self-rated health, with an alpha value of 0.05 and a power of 80% [39]. Hence, the computed sample size was 340 women residents after adjusting for a 20% non-response rate.

### Sampling techniques

A paper-form questionnaire was distributed to all women residents who agreed to participate in the study during door-to-door sample recruitment. A total of 1580 residential units were surveyed, and 420 women residents agreed to participate in the study. However, only 350 respondents fulfilled the criteria of a history of self-reported illness in the past three months and household income of ≤ RM9149.00; they were included in the sampling frame. Simple random sampling was performed from the sampling frame using an online random number generator accessed via calculatorsoup.com to pick the sampling unit until the targeted sample size reached 340.

### Measurements

The research instrument for this study was a printed self-administered questionnaire. Some of the questions were adopted and adapted from previous studies [34, 40–42]. The questionnaire was prepared in English, then translated and back-translated into Malay Language using the WHODAS 2.0 Translation Package [43]. The questionnaire was divided into two sections. Section I consisted of two screening questions aimed at detecting any reported illnesses in the past three months and the respondent’s household income. Section II consisted of six parts: Part A included questions on the sociodemographic and socioeconomic characteristics of the respondents; Part B was questions about the respondents’ health-seeking behaviours; Part C focused on questions regarding medical conditions and women’s autonomy in decision-making; Part D covered social support; Part E explored perceived stigma; and Part F examined attitudes toward health.

**Part A (Sociodemographic and Socioeconomic Characteristics).** This part had eight questions on sociodemographic and socioeconomic information. Sociodemographic information included age, ethnicity, education level, and marital status. While socioeconomic information encompassed employment status, monthly household income, and healthcare coverage. Sociodemographic and socioeconomic characteristics were measure using adapted questionnaire from the National Health and Morbidity Survey 2019 (NHMS 2019) [18] **Part B (Health-Seeking Behaviour).** On health-seeking behaviour, the questions were adapted from the NHMS 2019 [18] comprising of two items to assess the HSB of the respondents after they had experienced any of the 22 listed health problems in the last three months. **Part C (Medical Conditions and Women’s Autonomy in Decision-Making).** There were three questions in this part regarding respondents’ pre-existing chronic diseases, self-rated health and women’s autonomy in making decisions on health. Pre-existing chronic diseases and self-rated health were measured using questions adapted from NHMS 2019 [18] while women’s autonomy in decision-making was measured using a question adapted from Rani and Bonu [41]. **Part D (Social Support).** Social support was measured using an adapted questionnaire by Zimet et al. [42] that evaluated 12 items from three sources of support which were family, friends and special person. Respondents were required to rate a source of social support on a 7-point Likert scale. In this study, social support was classified into three categories which were strong social support (score between 5.1 and 7), moderate social support (score between 3 and 5), and weak social support (scores ranging from 1 to 2.9) [42]. **Part E (Perceived Stigma).** Perceived stigma was measured using an adapted questionnaire from the Stigma Scale for Receiving Psychological Help [40]. Respondents had to rate their agreement or disagreement with each statement on this 5-item scale using a 5 Likert-type scale. For this study, the level of perceived stigma was categorised into high perceived stigma (score ≥ 11) and low perceived stigma (score < 11). **Part F (Attitude Toward Health).** Attitude towards health was measured using an adapted questionnaire by Onyango et al. [34]. Respondents’ attitudes were measured using six items where they were required to indicate their views regarding illness treatment duration, treatment effects on marriage and work, community perception of ill patients and how they acquired the illness. The format of the questionnaire was a 5-Likert scale. For this study, attitude towards health was categorised based on mean scale scores which were positive attitude (score ≤ mean) and negative attitude (score > mean).

### Operational definition

The outcome of this study was health-seeking behaviour during times of illness. HSB was categorised into sought care and not sought care. Sought care is defined as respondents who had at least one visit to get advice or treatment on the listed illnesses for the past three months from any healthcare provider including a medical officer in a government health clinic, private clinic, and Accident and Emergency services in a public or private hospital.

The independent variables for this study were sociodemographic characteristics (age, education level, marital status, ethnicity), socioeconomic characteristics (employment status, household income, and healthcare coverage), medical conditions (pre-existing chronic diseases and self-rated health), women’s autonomy in decision-making, social support, perceived stigma and attitude toward health.

#### Data management and analysis

The data of this study were analysed using the Statistical Package for Social Sciences System (SPSS) version 28.0 software. The data were checked and cleaned before analysis was done. Descriptive statistics which were frequencies, percentages, means with standard deviations, and medians with interquartile ranges were calculated to describe the distribution of the variables. All the variables in this study showed high reliability, with Cronbach alpha values of 0.8 and above. On the other hand, Cohen’s Kappa test for self-rated health, pre-existing chronic diseases and women’s autonomy in decision-making showed values of 0.829, 0.841, and 0.761 respectively for this study.

All the numerical data were transformed into categorical data, such as age in years, monthly household income, social support, perceived stigma, and attitude towards health. Subsequently, Chi-square tests and Simple Logistic Regression analysis were conducted to measure the association between HSB during times of illness and independent variables. In this study, a significance level of 0.05 (*p* < 0.05) with a confidence interval of 95% was set.

Multiple logistic regression was performed to ascertain the predictors for sought care during times of illness among the respondents. Factors that were found to have a p-value of 0.25 in bivariate analysis were included for further multivariable analysis. Multiple logistic regression was done using three methods which were the “Enter” method, “Backward LR” method and “Forward LR” method. From these variables’ selection methods, the “Backward LR” method was used in the final model as it was the most parsimonious model that fitted the data well. Multiple logistic regression was conducted to determine the predictors of sought care, and the results were expressed as adjusted odds ratios with a 95% confidence interval not including one that was assumed significant.

## Results

### Characteristics of the participants

A total of 340 women residents participated in this study. The participants’ mean age was 44.51 ± 11.083 years, ranging from 18 to 59 years old. Most of the participants were Malay (81.8%), married (80.3%) and had secondary education (70.9%). The respondents’ mean household income was RM2649.47 ± 1367.185, which ranged from RM120 to RM9000. The majority of the respondents were housewives (50.6%), had an income of more than RM2216 (51.8%), and self-paid for their healthcare coverage (58.8%). Most of the respondents had no pre-existing chronic diseases (59.4%) and rated their health as good (50.3%). The majority of the respondents can make their own health decisions and have higher autonomy in decision-making (78.2%). Regarding social support, the majority of the respondents (66.8%) had strong social support with a mean score of 5.55 ± 0.797. The mean total score for perceived stigma was 10.78 ± 4.242 with most of the respondents (52.6%) having low perceived stigma. The mean score for attitude toward health was 2.65 ± 0.713 with 50.3% or 171 of the respondents had a negative attitude toward health (Table 1).

**Table 1:** Characteristics and prevalence of health-seeking behaviour during times of illness of the respondents (*N* = 340)

Characteristic	Mean ± SD	*n*	(%)
**Sociodemographic Characteristics**			
***Age (Years)***	44.51 ± 11.083		
18 to 25 years old		31	(9.1)
26 to 45 years old		134	(39.4)
> 45 years old		175	(51.5)
***Ethnicity***			
Malay		278	(81.8)
Chinese		12	(3.5)
Indian		50	(14.7)
***Marital status***			
Single		32	(9.4)
Married		273	(80.3)
Divorced		17	(5.0)
Widowed		18	(5.3)
***Education level***			
No formal education		8	(2.4)
Primary education		42	(12.4)
Secondary education		241	(70.9)
Tertiary education		49	(14.4)
**Socioeconomic Characteristics**			
***Employment status***			
Government servant		17	(5.0)
Private sector		82	(24.1)
Self-employed		48	(14.1)
Housewife		172	(50.6)
Student		20	(5.9)
Unemployed		1	(0.3)
***Household income (RM)***	2649.47 ± 1367.185		
Lower income (≤ RM2216)		164	(48.2)
Higher income (> RM2216)		176	(51.8)
***Healthcare coverage***			
Government employee’ GL /pensioner card		32	(9.4)
Government health funding program		23	(6.8)
Personal health insurance		8	(2.4)
Employer-sponsored insurance		6	(1.8)
Panel clinic/hospital		64	(18.8)
Self-paid		200	(58.8)
Spouse		1	(0.3)
Other family members		6	(1.8)
**Medical Conditions**			
***Pre-existing chronic diseases***			
Yes		138	(40.6)
No		202	(59.4)
***Self-rated health***			
Good		171	(50.3)
Fair		147	(43.2)
Poor		22	(6.5)
***Women’s autonomy in decision-making***			
Higher autonomy		266	(78.2)
Moderate autonomy		60	(17.7)
Lower autonomy		14	(4.1)
***Social support***	5.55 ± 0.797		
Strong social support		227	(66.8)
Moderate social support		113	(33.2)
Weak social support		0	(0)
***Perceived stigma***	10.78 ± 4.242		
Low		179	(52.6)
High		161	(47.4)
***Attitude toward health***	2.65 ± 0.713		
Positive attitude		169	(49.7)
Negative attitude		171	(50.3)
***Health-seeking behaviour***			
Self-medicate		37	(10.9)
Seek treatment at government health clinic		124	(36.5)
Seek treatment at private clinic		80	(23.5)
Seek treatment at A&E government hospital		42	(12.4)
Seek traditional care		15	(4.4)
Do nothing		42	(12.4)
***Daily activities disruption during times of illness***			
Yes		136	(40.0)
No		191	(56.2)
Not sure		13	(3.8)
***Health-seeking behaviour during times of illness***			
Sought care		246	(72.4)
Not sought care		94	(27.6)

SD: Standard deviation; GL: guarantee letter

### Health-seeking behaviour during times of illness

The prevalence of the respondents who sought care during times of illness was 72.4%, with the majority of the respondents reporting seeking treatment at a government health clinic (36.5%) and that no daily activities were disrupted during their illness episodes (56.2%) (Table 1).

### Predictors of sought care during times of illness

Based on chi-square analysis, HSB during times of illness was significantly associated with ethnicity (χ^2^ = 10.142, df = 1, *p* = 0.001), household income (χ^2^ = 6.690, df = 1, *p* = 0.011), healthcare coverage (χ^2^ = 11.707, df = 1, *p* < 0.001), pre-existing chronic diseases (χ^2^ = 4.053, df = 1, *p* = 0.044) and self-rated health (χ^2^ = 7.479, df = 1, *p* = 0.006), and women’s autonomy in decision-making (χ^2^ = 16.916, df = 2, *p* < 0.001) (Table 2).

**Table 2:** Factors associated with health-seeking behaviour during times of illness (*N* = 340)

Variable	Health-seeking behaviour during times of illness		χ^2^	df		*p*-value	
	Sought care *n* (%)	Not sought care *n* (%)					
**Sociodemographic Characteristics**							
**Age**							
18 to 25 years old	20 (64.5)	11 (35.5)	2.098	2		0.350	
26 to 45 years old	102 (76.1)	32 (23.9)					
≥ 46 years old	124 (70.9)	51 (29.1)					
**Ethnicity**							
Malay	191 (68.7)	87 (31.3)	10.142	1		0.001*	
Non-Malay	55 (88.7)	7 (11.3)					
**Education level**							
Lower education	212 (72.9)	79 (27.1)	0.252	1		0.616	
Higher education	34 (69.4)	15 (30.6)					
**Marital status**							
Single	19 (59.4)	13 (40.6)	2.981	2		0.225	
Married	201 (73.6)	72 (26.4)					
Widow/ divorcee	26 (74.3)	9 (27.6)					
**Socioeconomic Characteristics**							
**Employment status**							
Not working	140 (72.5)	53 (27.5)	0.008	1		0.930	
Working	106 (72.1)	41 (27.9)					
**Household income**							
Lower income	108 (65.9)	56 (34.1)	6.690	1		0.011*	
Higher income	138 (72.4)	38 (21.6)					
**Healthcare coverage**							
Yes	110 (82.7)	23 (17.3)	11.707	1		< 0.001*	
No	136 (72.4)	71 (34.3)					
**Medical Conditions**							
**Pre-existing chronic diseases**							
Yes	108 (78.3)	30 (21.7)	4.053	1		0.044*	
No	138 (68.3)	64 (31.7)					
**Self-rated health**							
Good	135 (78.9)	36 (21.1)	7.479	1		0.006*	
Fair/ poor	111 (65.7)	58 (34.3)					
**Women’s autonomy in decision-making**							
Higher autonomy	206 (77.4)	60 (22.6)	16.916	2		< 0.001*	
Moderate autonomy	34 (56.7)	26 (43.3)					
Lower autonomy	6 (42.9)	8 (57.1)					
**Social support**							
Strong	159 (70.0)	68 (30.0)	1.820	1		0.177	
Moderate	87 (77.0)	26 (23.0)					
**Perceived stigma**							
Low	129 (72.1)	50 (27.9)	0.015	1		0.901	
High	117 (72.7)	44 (27.3)					
**Attitude toward health**							
Positive	129 (76.3)	40 (23.7)	2.659	1		0.103	
Negative	117 (68.4)	54 (31.6)					

*Note: significant level at p < 0.05

Simple logistic regression revealed that ethnicity, marital status, household income, healthcare coverage, pre-existing chronic diseases, self-rated health, women’s autonomy, social support, and attitude toward health were included in the preliminary model of multiple logistic regression at *p* ≤ 0.25 (Table 3).

Ethnicity, healthcare coverage, self-rated health and pre-existing chronic diseases were significantly associated with HSB during times of illness in multiple logistic regression analyses (*p* < 0.05). Non-Malay respondents were 4.33 times more likely to seek care during illness than Malay respondents (95% CI: 1.847, 10.161, *p* < 0.001). Those respondents with healthcare coverage were 2.60 times more likely to seek care than those with no healthcare coverage (95% CI: 1.466, 4.612, *p* = 0.001). Respondents who rated their health as good were 1.87 times more likely to seek care than those who rated it as fair or poor (95% CI: 1.119, 3.118, *p* = 0.017). Nevertheless, respondents with pre-existing chronic diseases were recorded to be 1.92 times more likely to seek care during illness than those without pre-existing chronic diseases (95% CI: 1.130, 3.271, *p* = 0.016).

**Table 3:** Predictors of sought care during times of illness among participants

Variable	COR (95% CI)	*p*-value	AOR (95% CI)			*p*-value
**Ethnicity**						
Malay	**Reference**		**Reference**			
Non-Malay^†^	3.58 (1.566–8.178)	0.002*	4.33 (1.847–10.161)			< 0.001*
***Marital status***						
Single	**Reference**		**Reference**			
Married	1.91 (0.898–4.064)	0.093	1.30 (0.556–3.018)			0.549
Divorcee/ widow	1.98 (0.702–5.567)	0.197	1.09 (0.335–3.523)			0.890
***Household income***						
Lower income	**Reference**		**Reference**			
Higher income	1.88 (1.162–3.052)	0.010*	1.61 (0.955–2.727)			0.074
***Healthcare coverage*** ^***††***^						
No	**Reference**		**Reference**			
Yes	2.50 (1.465–4.255)	< 0.001*	2.60 (1.466–4.612)			0.001*
***Self-rated health***						
Fair/ poor	**Reference**		**Reference**			
Good	1.96 (1.205–3.185)	0.007*	1.87 (1.119–3.118)			0.017*
***Pre-existing chronic diseases***						
No	**Reference**		**Reference**			
Yes	1.67 (1.011–2.757)	0.045*	1.92 (1.130–3.271)			0.016*
***Women’s autonomy in decision-making***						
Lower autonomy	**Reference**		**Reference**			
Moderate automony	1.74 (0.538–5.647)	0.354	1.12 (0.317–3.943)			0.863
Higher autonomy	4.58 (1.529–13.709)	0.007*	2.06 (0.612–6.927)			0.243
***Social support***						
Moderate support	**Reference**		**Reference**			
Strong support	0.70 (0.415–1.178)	0.178	0.67 (0.363–1.179)			0.158
***Attitude toward health***						
Negative	**Reference**		**Reference**			
Positive	1.49 (0.922–2.404)	0.104	1.19 (0.697–2.023)			0.527

COR: crude odds ratio, AOR: adjusted odds ratio, CI: confidence interval

^***†***^Non-Malay (Chinese & Indian) ^***††***^Healthcare coverage: Yes (government employee’ GL/pensioner card, government health funding program, personal health insurance, employer-sponsored health insurance, and panel clinic)

*Statistical significance at *p* < 0.05

Backward LR variables selection method was applied

Multicollinearity (Variance-inflation factor < 10) and interaction terms were checked

Hosmer and Lameshow test (*p* = 0.257), classification table (overall percentage: 73.8%), Cox & Snell R square (0.117), Nagelkerke R square (0.169), ROC curve: 71.3%

## Discussion

In this study, most respondents (72.4%) sought care during times of illness. Compared to previous studies, the prevalence of HSB in this study was much higher than reported among women in developed countries such as Korea [44] and Norway [8] and other developing countries such as India [45] and Cambodia [46]. For local studies, the prevalence of HSB ranged between 23.1 and 85.9% with urban areas showing a higher prevalence of HSB as compared to rural areas [47, 48]. This could probably be due to the availability and accessibility of better healthcare facilities in urban areas [28]. In Malaysia, the average distance of government health clinic coverage was 9.71 km while private clinic coverage was 0.472 km with a ratio of health clinics to population at 1:4228 [49, 50]. In urban regions of Malaysia, there is a low density of government health clinics relative to the population, a gap compensated by the clustering of private clinics. Conversely, rural areas exhibit a higher concentration of government health clinics, a strategic response to address challenges related to geographical accessibility [54]. The healthcare system in Malaysia is characterised by significant subsidisation, with consultation and medication fees set at only RM1 across all public health clinics for citizens, while children under five and the elderly receive these services free of charge. Despite this, numerous private health clinics in urban areas do not effectively address the health issues of urban poor women due to financial constraints. In recognition of this disparity, the Ministry of Health is actively tackling the matter by developing alternative healthcare facilities with a narrower range of services administered only by paramedics. These facilities primarily focus on minor ailments and simple procedures [51].

The majority of the respondents in the present study sought care from healthcare facilities. In contrast, respondents who did not seek care during illness either self-medicated (10.9%), took no action (12.4%), or sought traditional care (4.4%). These findings are comparable with findings from a population survey done in Malaysia [18]. With the advancement of technology and widespread internet access, information can be shared just at the tip of the finger, whether the information is legitimate or needs to be pondered. A study done by Fox and Duggan showed that 35% of internet users had used this online platform to find information related to health, post health-related questions or share their experiences related to health [52]. This “online health seekers” trend influenced many self-medicated practices [18]. Despite the extensive distribution of conventional medicine, traditional and complementary medicine (T&CM) is still one of the choices for Malaysians to support their health, as they believe the treatment is effective [53]. TCM is commonly used to treat both communicable and non-communicable diseases, especially chronic diseases including cancer, diabetes, and rheumatoid arthritis [54]. A large body of research underlines that many consumers perceive T&CM as a safer option to conventional medicine, and the accessibility and perceived effectiveness of natural approaches emerge as major variables affecting the preference for T&CM in promoting health [55]. Moreover, studies further reveal that those with insufficient health literacy are more prone to use T&CM [56]. Following the establishment of the National Traditional and Complementary Medicine Division in Malaysia, T&CM is being integrated into certain government hospitals to provide selected T&CM practices such as herbal therapy for adjuvant treatment of cancer and postnatal care, as well as traditional massage and acupuncture for post-stroke management. As a result, the satisfaction gained from combining T&CM with conventional medication reflects the need not to limit the use of conventional medicine [54].

Similar to findings from a study done among low-income women in California, HSB was significantly associated with ethnicity [57]. This could contribute to the observed differences in health-seeking behaviour among different ethnicities. Household income and women’s autonomy in decision-making were significantly associated with HSB in our study and are similar to findings from Ethiopia and India [24, 41]. A study in Ghana showed healthcare coverage was significantly associated with accessing healthcare [58] which was similar to the finding of this study. Pre-existing chronic diseases were also identified as a significant factor of HSB in our study, similar to the findings of previous studies done in Ethiopia, Greece and India [59–61]. A study done in Uganda found that self-rated health was significantly associated with HSB [62], a similar finding with our study.

In this study, it was found that being non-Malay was a significant predictor of HSB during times of illness. Non-Malays were four times more likely to seek care during illness than Malay respondents. Many studies have been undertaken in Malaysia to establish the relationship between ethnicity and HSB, but no significant association has been identified [28, 47]. However, studies reveal that being Malay increases the likelihood of seeking traditional medicine compared to being non-Malay [63, 64]. In Malaysia, ethnicity significantly shapes health-seeking behaviour, particularly among marginalised populations such as urban poor women. Socioeconomic and cultural factors and systemic inequities often contribute to differential healthcare utilisation patterns among different ethnic groups and Malay ethnicity was more likely to utilise complementary and alternative medicine as compared to other ethnicity [64].

Having healthcare coverage such as health insurance, pensioner cards, government employees’ guarantee letters, and government health funding programs made people three times more likely to seek healthcare during times of illness. Studies have consistently shown that women with healthcare coverage are more likely to seek preventive services, timely screenings, and treatment when needed [58, 65, 66]. This increased utilisation of healthcare services is crucial for early detection, prevention, and management of health conditions, leading to improved health outcomes. By increasing the chances of going for a medical check-up and early treatment, health insurance can save costs on curative health [65]. Financial problems such as poverty, financial dependence, and the high cost of services, identified as the main barriers to accessing healthcare, can be counter-measured by having healthcare coverage [67].

In this study, urban poor women who rated their health as good were two times more likely to seek care than those who rated it as fair or poor. Self-rated health reflects an individual’s subjective assessment of their overall health status, incorporating physical, mental, and social well-being [68]. This perception can influence HSB among urban poor women in several ways. Firstly, individuals with positive or good self-rated health tend to engage in preventive health behaviours such as regular check-ups, screenings, and maintaining a healthy lifestyle [69]. They are more likely to seek healthcare services as a proactive measure to maintain and improve their well-being. They tend to be more health-conscious and motivated to engage in health-promoting behaviours [70, 71]. Conversely, those with negative or poor self-rated health may delay seeking care or only seek it when their health deteriorates significantly or when they experience functional limitations [72], which are associated with unmet healthcare needs [62].

Lastly, respondents with pre-existing chronic diseases were two times more likely to seek care than those without. People with chronic diseases often require regular medical attention and monitoring to manage their conditions effectively. When they experience illness or an exacerbation of their chronic condition, promptly seeking medical care becomes a priority [73]. Chronic diseases can make individuals more vulnerable to infections or complications. Consequently, women with pre-existing chronic diseases may perceive themselves at higher risk and seek care more readily when they become ill to avoid long-term impacts on their health and mortality [74]. Individuals with chronic diseases may interact regularly with healthcare providers to manage their healthcare needs [9], with various health education activities given on each visit. This ongoing relationship can facilitate access to healthcare and increase their likelihood of seeking care during illness episodes.

### Strengths and limitations of the study

The study was carried out in a residential area designated for a low-income population and used a population-based approach, which is thought to be superior to an institutional-based study. The data was collected face-to-face through the distribution of printed questionnaires, and any uncertainties or lack of comprehension about the questions could be addressed by directly consulting the researcher in the field, resulting in a 100% response rate and reducing missing data.

However, this study was a cross-sectional study, therefore, the causal-temporal relationship cannot be determined between HSB during times of illness and independent variables. This study also did not address other factors, such as health service accessibility, or use any theoretical framework as the study background. One of the variables, women’s autonomy in decision-making, used only one question to measure the variable, which may not provide enough information or accurately measure the variable. Besides, recall bias may arise in this study as the respondents must self-report whether they have experienced any of the listed health problems or report their actions during the illness period. In addition, the generalisation of the study findings may be limited because this study was conducted in a people’s housing project within Kuala Lumpur and might not represent the other urban poor women in other populations in Malaysia and other populations with different sociodemographic backgrounds.

### Recommendations

Given that 72.4% of the respondents sought care during times of illness, there is still a need to further improve the HSB among urban poor women. Besides, other factors such as health services accessibility associated with HSB in urban poor women should have been studied. Qualitative studies, such as in-depth interviews or focus group discussions, should be conducted to better understand the factors influencing health-seeking behaviour among urban poor women. It can provide insights into the specific barriers and facilitators they face and the cultural and social factors that may influence their decision-making. Thus, other variables should be explored in the future to determine the association with HSB. Expanding the research to include a larger sample size of urban poor populations in Malaysia with different sociodemographic backgrounds should also be considered. Studies could also include urban poor women who do not reside in a people’s housing project housing types and involve multiple districts or states to improve the problems by generalising the study findings.

## Conclusions

This study was conducted to determine the predictors of HSB during times of illness among urban poor women and discovered that HSB during times of illness among the participants was appropriate. The study has identified ethnicity, healthcare coverage, self-rated health, and pre-existing chronic diseases as predictors of HSB during times of illness.

Health promotion and targeted health education should be given through an individual or community approach, to those of Malay ethnicity, individuals with fair or poor self-rated health, no healthcare coverage and without pre-existing chronic diseases, focusing on education and raising awareness about the importance of seeking timely healthcare. Collaboration with relevant stakeholders, such as local authorities, non-governmental organisations and community leaders, is needed to develop comprehensive strategies that address the multifaceted challenges faced by urban poor women in accessing and seeking healthcare services.

## Data Availability

The dataset for the current study is available from the corresponding author upon receipt of a reasonable request.

## Abbreviations

HSB: Health-seeking behaviour
UHC: Universal health coverage
SQA: Screening question A
SQB: Screening question B
CI: Confidence interval
SD: Standard deviation
AOR: Adjusted odds ratio
GL: Guarantee letter
T&CM: Traditional and complimentary medicine
